# Authors’ response: Atypical age distribution and high disease severity in children with RSV infections during two irregular epidemic seasons throughout the COVID-19 pandemic

**DOI:** 10.2807/1560-7917.ES.2024.29.20.2400282

**Published:** 2024-05-16

**Authors:** Wei Cai, Sophie Köndgen, Kristin Tolksdorf, Ralf Dürrwald, Barbara Biere, Walter Haas, Thorsten Wolff, Silke Buda, Janine Reiche

**Affiliations:** 1 Unit 36, Respiratory Infections, Department of Infectious Disease Epidemiology, Robert Koch Institute, Berlin, Germany; 2 Unit 17, Influenza and Other Respiratory Viruses, Department of Infectious Diseases, Consultant Laboratory for RSV, PIV and HMPV, Robert Koch Institute, Berlin, Germany; 3 Unit 17, Influenza and Other Respiratory Viruses, Department of Infectious Diseases, National Influenza Centre, Robert Koch Institute, Berlin, Germany

**Keywords:** respiratory syncytial virus, surveillance, genotype, acute respiratory infection, disease severity, children

**To the editor:** Based on our well-established, long-term national virological and syndromic sentinel surveillance systems in primary and secondary healthcare, we recently reported that during the COVID-19 pandemic, both the 2021 and 2022/23 respiratory syncytial virus (RSV) seasons started earlier and showed increased virus circulation compared with the pre-COVID-19 seasons in Germany [1]. Furthermore, children aged 2–4 years were at high risk of RSV infection in 2021, while in the 2022/23 season, the severity of RSV disease increased, with a higher proportion of children, especially those aged 0–3 months, requiring intensive care treatment and ventilator support. We suggested that these observations could be explained by a lower baseline immunity in the population after non-pharmaceutical interventions (NPIs) reduced exposure to RSV during the COVID-19 pandemic. The letter from Liu et al. [2] provides some thoughtful comments, but challenges this hypothesis based upon assumptions that we would like to put into perspective.

First, it is discussed whether the increased severity of RSV disease in 0–3-month-olds in the 2022/23 RSV season was still due to reduced baseline population immunity resulting from anti-epidemic measures. In fact, a variety of NPIs including mandates to wear masks, closures of schools and childcare facilities, restaurants and retail, or testing regimes were introduced in Germany in 2020 to control the spread of severe acute respiratory syndrome coronavirus 2 (SARS-CoV-2). While some of the NPIs were lifted in 2021, others continued until March 2023 in Germany [1,3]. In addition, a serological survey conducted in the Netherlands between June 2020 and June 2021 provided evidence that RSV-specific immunoglobulin (Ig)G levels decreased in all age groups when RSV circulation was limited or absent [4]. Considering that NPIs were not fully lifted in Germany until March 2023, the most probable scenario is that even the strong 2021 RSV season did not fully restore basic immunity in the German population including that of pregnant women who transfer protective maternal antibodies to their infants. For the 0–3-month-olds, we therefore suggest that a larger proportion of the newborns in the 2022/23 season had insufficient immune protection against RSV, leading to increased severity as the most likely explanation. It remains to be determined whether the alternating dominance of RSV-A and RSV-B subgroups in the two seasons, which may have reduced baseline population immunity to RSV, also contributed to this situation.

Second, Liu et al. stated that our study did not address possible changes in RSV testing before and after the COVID-19 pandemic that may have overestimated RSV detections during the 2021 and 2022/23 RSV seasons. There was certainly a general increase in testing for respiratory viruses during the pandemic. However, there are several lines of evidence suggesting that the data reported in our study reflect a true increase in the incidence and/or severity of RSV disease in children: As shown in Table 1 of our study, the outpatient and inpatient data come from two sentinel systems, which have been in place for several years, sampling representative proportions of the population. There was no change in data and sample collection, nor any relevant change in case definition throughout the study period [1]. In addition, since 2011/12, all primary care samples have been continuously tested for RSV as part of the virological surveillance using sensitive, quality-assured in-house PCR systems, thereby allowing the extent of RSV circulation in the population to be assessed in terms of positivity rates. Beyond that, in secondary healthcare, a standard diagnostic procedure for young children was established before the COVID-19 pandemic in the paediatric units of hospitals participating in our sentinel syndromic surveillance. Suspected cases of RSV in young children are tested, and laboratory-confirmed RSV infections are coded with RSV-specific ICD-10 codes [5]. Thus, the COVID-19 pandemic did not change standard diagnostic practice in our primary virological or syndromic secondary healthcare surveillance systems. We believe, the fact that the healthcare surveillance systems in Germany were established as year-round surveillance for several years has been an advantage in collecting structured and sustainable population-based public health data and in reliably monitoring the RSV circulation and disease burden of RSV in the context of the COVID-19 pandemic.

Third, although we suggest that the increases in RSV circulation during the 2021 to 2022/23 RSV seasons were mainly a consequence of reduced (humoral) population immunity due to anti-epidemic measures, we do not rule out a contribution from immune dysfunction following SARS-CoV-2 infection [6], as mentioned by Liu et al. [2]. The cellular immune states and conditions that affect the functionality of different arms of the immune system in patients with COVID-19 and their consequences for future infections are complex [7]. This broad topic was not addressed by our study, but clearly needs to be investigated in future comprehensive longitudinal clinical studies, in addition to continued monitoring of the circulation and evolution of respiratory viruses including RSV.
